# Novel *ACAD8* variants identified in Isobutyryl-CoA dehydrogenase deficiency: challenges in phenotypic variability and management

**DOI:** 10.3389/fgene.2025.1532902

**Published:** 2025-04-22

**Authors:** Yilun Tao, Dong Han, Jianfang Li, Xiaoyun Li, Luna Hao, Wenxia Song, Lihong Wang, Xiaoze Li

**Affiliations:** ^1^ Precision Medicine Research Division, Changzhi Maternal and Child Healthcare Hospital, Changzhi, Shanxi, China; ^2^ Medical Genetic Center, Changzhi Maternal and Child Healthcare Hospital, Changzhi, Shanxi, China; ^3^ Department of Pediatrics, Changzhi Maternal and Child Healthcare Hospital, Changzhi, Shanxi, China

**Keywords:** *ACAD8* gene, isobutyryl-CoA dehydrogenase deficiency, newborn screening, next-generation sequencing, novel variant

## Abstract

Isobutyryl-CoA dehydrogenase deficiency (IBDD) is a rare autosomal recessive disorder caused by biallelic variants in the *ACAD8* gene, which disrupts valine metabolism. In this study, we report seven individuals identified through newborn screening (NBS) with elevated C4-acylcarnitine levels, including five confirmed patients and two heterozygous carriers. Genetic analysis identified 12 distinct *ACAD8* variants, seven of which were novel (c.221C>T, c.518T>C, c.727A>G, c.868G>A, c.947A>T, c.966G>A, c.1058T>C). According to ACMG classification criteria, c.221C>T was classified as likely pathogenic, while the remaining variants were categorized as variants of uncertain significance (VUS). During a mean follow-up of 4.81 years, all patients maintained normal growth patterns but two patients developed neurological symptoms that included recurrent febrile seizures and sensory integration dysfunction. These findings expand the *ACAD8* variant spectrum, highlight the phenotypic variability of IBDD, and underscore the importance of long-term follow-up and individualized management strategies.

## 1 Introduction

Isobutyryl-CoA Dehydrogenase Deficiency (IBDD) is a rare autosomal recessive metabolic disorder with diverse clinical manifestations. While most individuals with IBDD are asymptomatic, a subset may develop significant clinical symptoms, including dilated cardiomyopathy, anemia, and systemic carnitine deficiency, which can lead to developmental complications if untreated (Oglesbee et al., 2007). Some affected individuals have presented with muscle hypotonia, mild developmental delays, and speech impairments during infancy (Koeberl et al., 2003; Sass et al., 2004; Pedersen et al., 2006). Early detection and intervention, particularly with oral carnitine supplementation, are crucial in preventing progression and optimizing long-term outcome (Knerr et al., 2012; Santra et al., 2017; Feng et al., 2021).

First described by Roe et al., in 1998 (Roe et al., 1998), IBDD was later linked to pathogenic variants in *ACAD8* gene (MIM 604773), located on chromosome 11q25 (Nguyen et al., 2002). The *ACAD8* gene encodes isobutyryl-CoA dehydrogenase, a mitochondrial enzyme essential for the third step in valine degradation. Enzymatic dysfunction results in the accumulation of isobutyryl-CoA and elevated C4-acylcarnitine, which can be detected via tandem mass spectrometry (MS/MS) in newborn screening (Pedersen et al., 2006).

IBDD is an extremely rare disorder, with estimated prevalence rates ranging from approximately 1 in 30,000 to 1 in 60,000 live births (Feng et al., 2021; Zhuang et al., 2021). The rarity of the disorder, combined with its highly variable phenotype, often leads to misdiagnosis or delayed recognition, complicating clinical management. Advances in MS/MS technology have facilitated the identification of IBDD by detecting characteristic biochemical markers, while next-generation sequencing (NGS) enables definitive genetic confirmation.

This study aims to identify novel ACAD8 variants in patients with elevated C4-acylcarnitine levels detected through newborn screening, thereby expanding the known variant spectrum of IBDD, enhancing the understanding of its clinical heterogeneity, and providing deeper insights into the disease’s natural course.

## 2 Materials and methods

### 2.1 Subject and ethical statement

From May 2015 to April 2024, a total of 227,583 individuals underwent newborn screening using MS/MS at Changzhi Maternal and Child Healthcare Hospital. Newborns identified with elevated C4-acylcarnitine levels underwent confirmatory testing, including urine organic acid analysis using gas chromatography-mass spectrometry (GC/MS) and molecular analysis via NGS. The study was approved by the Ethics Committee of Changzhi Maternal and Child Healthcare Hospital. Written informed consent was acquired from the parent or legal guardian of the patient before commencing the study.

### 2.2 Tandem mass spectrometry analysis

Dried blood spot (DBS) samples, each with a diameter greater than 8 mm, were collected from each participant within the first 48 h of life as part of the newborn screening protocol. Acylcarnitine profiles were analyzed via MS/MS using the NeoBase™ non-derivatized MS/MS kit (PerkinElmer, United States) on an ACQUITY TQD mass spectrometer (Waters, Milford, MA, United States). A 3.2 mm DBS punch was placed into a 96-well microplate, and 100 µL of extraction buffer containing isotopically labeled internal standards was added. The plate was incubated at 45°C for 45 min under continuous orbital agitation (700 rpm) to facilitate metabolite extraction. After centrifugation (1,500 × g, 5 min), 75 µL of supernatant was transferred to a clean plate and left to equilibrate at ambient temperature for 2 h before injection. Samples were analyzed in electrospray ionization (ESI) positive mode, with multiple reaction monitoring (MRM) transitions targeting specific acylcarnitines. Key instrument parameters included a capillary voltage of 3.5 kV, source temperature of 120°C, desolvation temperature of 350°C, cone gas flow of 20 L/h, and desolvation gas flow of 650 L/h. Target analytes (e.g., C4-acylcarnitine: m/z 232.1 → 85.1) were monitored (Supplemental Table S1), along with corresponding internal standards. Quantification was based on the ratio of analyte peak area to internal standard peak area. Quality control was ensured using two levels of internal standards representing low and high concentrations. A C4-acylcarnitine threshold of 0.45 μmol/L, established from the 99. fifth percentile of local population screening data, was used to flag samples for further diagnostic confirmation.

### 2.3 GC/MS analysis

Urinary organic acid analysis was performed using gas chromatography-mass spectrometry (GC/MS). Urine samples were collected either as dried urine filter paper specimens for external submissions or as fresh samples from patients treated at our hospital. The analysis was conducted using the GCMS QP-2010 Plus system (Shimadzu, Kyoto, Japan) following the manufacturer’s protocol (Aiwan, Shenzhen, China). A semi-quantitative approach was employed, utilizing a single reference standard to estimate target compound concentrations. Urine creatinine levels were first measured, and an aliquot corresponding to 0.2 mg of creatinine was used for analysis. The selected sample underwent urea removal, followed by an oximation reaction to stabilize keto acids. Organic acids were then extracted using ethyl acetate, and the extract was derivatized with a silylation reagent before GC/MS analysis. Heptadecanoic acid served as the internal standard, and metabolite concentrations were estimated based on the peak area ratio of the target compound to the internal standard.

### 2.4 Genetic analysis

Peripheral blood samples were collected from each patient for genetic analysis. Genomic DNA was extracted using the QIAamp DNA Mini Kit (Qiagen, China), following the manufacturer’s protocol. DNA library preparation was conducted in accordance with Illumina protocols, which included end repair, adapter ligation, and PCR enrichment. Library enrichment was performed using the whole exome capture kit (MyGenostics Inc., Beijing, China) with biotinylated capture probes targeting all exons, avoiding duplications. The enriched libraries were sequenced on the Illumina HiSeq × Ten platform with 150 bp paired-end reads. Following sequencing, sequencing data were processed and aligned to the human genome reference sequence (hg19) using the Burrows-Wheeler Alignment (BWA) tool, and duplicated reads were removed using Picard to enhance data accuracy. Variants were called using Genome Analysis Toolkit (GATK) and annotated through ANNOVAR. Variants were cross-referenced with databases including the 1000 Genomes Project, Exome Aggregation Consortium, GnomAD, and the Human Gene Mutation Database (HGMD). This study focused on nonsynonymous variants, splicing site variants within 10 bp of exon-intron boundaries, and clinically relevant splicing site variants, excluding synonymous and non-coding exonic variants. Pathogenicity assessments of identified variants followed the guidelines of the American College of Medical Genetics and Genomics (ACMG) (Richards et al., 2015). Sanger sequencing was used to validate identified variants in both the patients and their parents.

### 2.5 Biochemical and clinical follow-up

Patients carrying *ACAD8* variants underwent clinical follow-up to monitor potential symptoms of IBDD, including developmental assessments and metabolic profiles. C4-acylcarnitine levels were periodically measured using DBS.

## 3 Results

### 3.1 Patient demographics and biochemical findings

Among 227,583 individuals screened by MS/MS, 177 cases exhibited elevated C4-acylcarnitine levels. Among these cases, seven individuals exhibited elevated C4-acylcarnitine levels (mean: 1.30 μmol/L, range: 0.67–2.32 μmol/L) as detected by MS/MS (Table 1), with concurrent identification of variants in the *ACAD8* gene through molecular genetic testing. GC-MS analysis revealed increased isobutyrylglycine (IBG) in four cases and 3-hydroxypropionate (3-HP) in three cases. Ethylmalonic acid levels remained within the normal range in all cases. Based on genetic analysis, five individuals were confirmed as IBDD patients, while two were identified as heterozygous carriers, yielding an estimated regional incidence of 1 in 45,517. The cohort included seven individuals—five males and two females—with a mean age at diagnosis of 40.33 ± 2.58 days (range: 37–45 days). All individuals were asymptomatic at diagnosis.

**TABLE 1:** Summary of biochemical testing, genetic analysis, and clinical manifestations in seven cases.

Case	Gender	Allele 1	Allele 2	Pathogenicity	Testing time	MS/MS			GC/MS			Age at diagnosis	Report age (years)	Clinical manifestation
						C4 (0.04–0.45)	C4/C2 (0–0.04)	C4/C3 (0.02–0.33)	Lactate (0–4.7)	Isobutyrylglycine (0–0.4)	3-Hydroxypropionate (0–1.1)			
1	Female	c.409G>A	c.868G>A	P/VUS	4d	2.32	0.19	1.54	—	—	—	45d	7.25	Unremarkable
					17d	1.66	0.15	0.94	7.68	0.54	(−)			
					7y	1.74	0.13	1.05	—	—	—			
2	Male	c.221C>T	c.500del	LP/P	7d	1.45	0.10	0.98	—	—	—	41d	7	Unremarkable
					18d	1.02	0.06	0.39	(−)	0.42	(−)			
					7y	1.28	0.07	0.67	—	—	—			
3	Male	c.947A>T	c.1058T>C	VUS/VUS	4d	0.77	0.08	0.38	—	—	—	37d	5.33	Anemia (0.17y), sensory integration dysfunction (4.67y)
					20d	1.32	0.11	0.61	8.55	1.7	5.13			
					5y	1.08	0.07	0.6	—	—	—			
4	Female	c.512C>G	c.518T>C	VUS/VUS	3d	0.67	0.06	0.7	—	—	—	40d	3.08	Seizure and abnormal CT and EEG (1.08y); mild speech delay (3y)
					16d	0.67	0.05	1.06	(−)	(−)	(−)			
					3y	0.96	0.06	0.73	—	—	—			
5	Female	c.258C>G	c.1176G>T	LP/LP	3d	2.29	0.09	0.91	—	—	—	40d	0.92	Unremarkable
					13d′	1.51	0.13	1.27	(−)	(−)	(−)			
					0.5y	1.37	0.09	0.78	—	—	—			
6	Female	c.727A>G	-	VUS/-	3d	0.79	0.04	0.38	—	—	—	40d	5.83	Unremarkable
					22d	0.66	0.07	0.8	(−)	(−)	3.63			
					5.5y	0.52	0.04	0.32	—	—	—			
7	Female	c.966G>A	-	VUS/-	6d	0.82	0.05	0.74	—	—	—	40d	4.25	Anemia (0.083y)
					17d	0.54	0.07	0.89	(−)	0.61	1.16			
					4	0.46	0.05	0.34	—	—	—			

### 3.2 Genetic analysis

**FIGURE 1:**
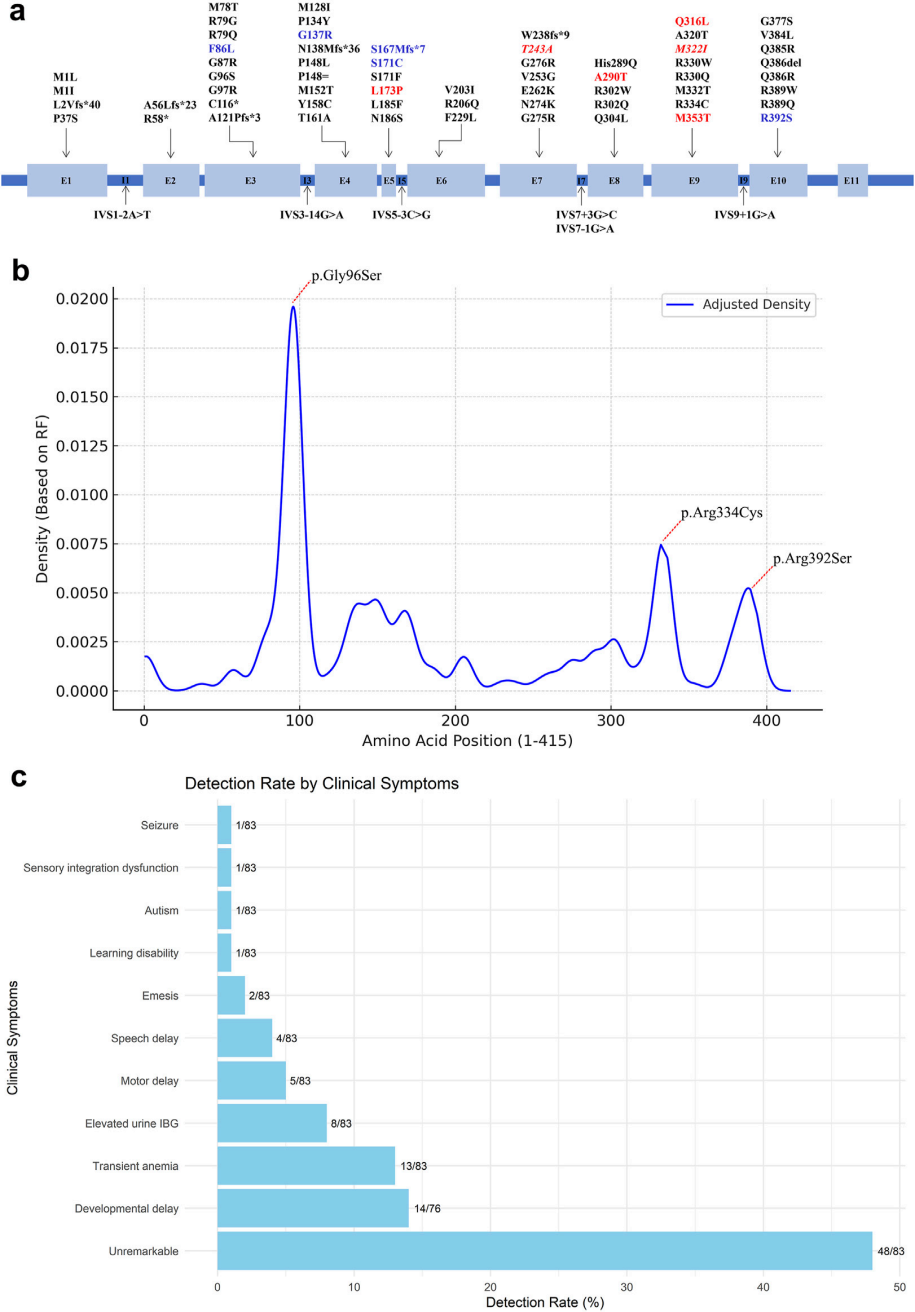
Overview of reported *ACAD8* gene variants. **(A)** Schematic illustration of the intron–exon structure of the *ACAD8* gene. Novel variants identified in this study are highlighted in red, while those detected in carriers are shown in red italics. Other variants identified in this study are marked in blue. **(B)** Mutation density plot along the *ACAD8* protein sequence, showing the distribution of variants at each amino acid position, weighted by their relative frequencies (RF%) using Kernel Density Estimation (KDE) in R version 4.3.0. **(C)** Frequency of clinical manifestations, represented as percentages and counts, arranged in ascending order.

NGS identified 12 distinct variants in the *ACAD8* gene (NM_014384.3) among the seven patients, including 11 missense variants and 1 frameshift variant (Figure 1A). Seven of these variants were novel and had not been previously reported. The novel variants included c.221C>T (p.Pro74Leu), c.518T>C (p.Leu173Pro), c.727A>G (p.Thr243Ala), c.868G>A (p.Ala290Thr), c.947A>T (p.Gln316Leu), c.966G>A (p.Met322Ile), and c.1058T>C (p.Met353Thr). These variants were distributed across exons 3, 4, 5, 7, 8, 9, and 10 of the *ACAD8* gene. According to ACMG guidelines (Richards et al., 2015), the novel variant p. Pro74Leu was classified as likely pathogenic, aligning with previously reported pathogenic variants such as p. Gly137Arg, p. Ser167MetfsTer7, p. Phe86Leu, and p. Arg392Ser (Supplemental Table S2). The remaining variants were categorized as variants of uncertain significance (VUS).

### 3.3 Treatment and follow-up

Except for Case 4, all patients received oral carnitine supplementation at a dose of 50 mg/kg/day, with no reported adverse effects. During a follow-up period averaging 4.81 years (ranging from 0.92 to 7.25 years), all patients demonstrated normal growth and developmental progress. However, several patients exhibited notable clinical manifestations:

Case 3: At 2 months of age, this patient exhibited signs of anemia, which resolved spontaneously within a few months without medical intervention. By 4 years and 8 months, the patient was diagnosed with sensory integration dysfunction, leading to difficulties in adapting to kindergarten. Motor and intelligence evaluations remained normal.

Case 4: At 1 year and 1 month old and again at 2 years and 4 months, the patient experienced three seizure episodes, triggered by COVID-19 infection and febrile illness. Cranial computed tomography (CT) revealed bilateral expansion of the subarachnoid space in the frontal, temporal, and parietal regions, along with enlargement of the fifth and sixth ventricles and the cerebellomedullary cistern. Electroencephalography (EEG) showed bilateral spike-wave and spike discharges at the frontal-central derivations. The patient also exhibits mild speech developmental delay and is currently undergoing orofacial rehabilitation therapy.

Case 7: This patient was diagnosed with mild anemia at 1 month old, as indicated by a reduced red blood cell (RBC) count (3.71 × 10^12^/L, reference: 4.0–5.0 × 10^12^/L) and a slightly decreased hematocrit (HCT) level (34.3%, reference: 35%–45%). No additional hematological investigations were conducted, and the anemia resolved naturally over time without requiring specific treatment.

## 4 Discussion and conclusions

To date, 66 distinct variants were identified in the *ACAD8* gene across 100 reported cases worldwide, comprising 50 missense, 6 frameshift, 6 splice-site, 2 nonsense, 1 small deletion, and 1 synonymous variant (Supplemental Table S3, S4) (Nguyen et al., 2002; Sass et al., 2004; Pedersen et al., 2006; Yoo et al., 2007; Oglesbee et al., 2007; Popek et al., 2010; Pena et al., 2012; Scolamiero et al., 2015; Yun et al., 2015; Lin et al., 2018; Wang et al., 2019; Lee et al., 2019; Navarrete et al., 2019; Sadat et al., 2020; Eleftheriadou et al., 2021; Feng et al., 2021; Zhuang et al., 2021; Tummolo et al., 2022; Liu et al., 2023; Men et al., 2023). In contrast to prior studies that identified recurrent variants such as c.286G>A (p.Gly96Ser) (25.5%), c.1000C>T (p.Arg334Cys) (9.00%), and c.1176G>T (p.Arg392Ser) (3.50%), which are predominantly reported in East Asian populations, none of these were found in our cohort (Figure 1B). Instead, seven novel variants were identified: c.221C>T (p.Pro74Leu), c.518T>C (p.Leu173Pro), c.727A>G (p.Thr243Ala), c.868G>A (p.Ala290Thr), c.947A>T (p.Gln316Leu), c.966G>A (p.Met322Ile), and c.1058T>C (p.Met353Thr). The absence of common hotspot variants and the discovery of novel variants reflect significant regional genetic heterogeneity, highlighting the unique genetic profile of the local cohort.

A striking feature of IBDD is its marked phenotypic variability, which challenges predicting clinical outcomes based on genotype alone (Lin et al., 2018; Feng et al., 2021; Zhuang et al., 2021). Among the 100 reported patients, 56.5% remained asymptomatic despite carrying biallelic *ACAD8* variants, underscoring the critical role of newborn screening (NBS) in identifying affected individuals who might otherwise go undiagnosed. However, a subset developed clinical manifestations, including developmental delay (14/76, 16.5%), transient anemia (13/83, 15.66%), elevated urine IBG (8/83, 9.64%), speech delay (4/83, 4.82%), and motor delay (5/83, 6.02%) (Figure 1C). Thirteen IBDD cases, including two from this study, have exhibited transient anemia (Knerr et al., 2012; Feng et al., 2021). However, a causal link between IBDD and anemia remains unestablished. In our cases, anemia resolved spontaneously without intervention, suggesting it may be incidental rather than a defining clinical feature of IBDD. Notably, asymptomatic individuals may transition to symptomatic states with age, presenting with late-onset complications such as fatigue, muscle weakness, and cardiomyopathy (Oglesbee et al., 2007; Zhuang et al., 2021). In our cohort, one patient developed sensory integration dysfunction, while another experienced febrile illness-triggered seizures, despite normal early development. Conversely, some patients remained asymptomatic, even with likely pathogenic or pathogenic variants (e.g., cases 2 and 5), underscoring the role of environmental influences, metabolic stress, and potential modifier genes in shaping disease expression. The lack of a robust genotype-phenotype correlation suggests that IBDD is driven by a complex interplay between intrinsic genetic factors and extrinsic metabolic demands, complicating prognostic predictions (Lin et al., 2018; Feng et al., 2021; Zhuang et al., 2021). Future research integrating genomic, transcriptomic, and metabolomic analyses is essential to identify modifying factors and refine risk stratification for IBDD patients.

L-carnitine supplementation is integral to IBDD management, facilitating the excretion of toxic isobutyryl-CoA intermediates and preventing secondary carnitine depletion (Nasser et al., 2012). In this study, six of seven patients received oral L-carnitine (50 mg/kg/day), with no reported adverse effects. All treated patients maintained normal growth and developmental milestones, further supporting the safety and therapeutic benefit of carnitine supplementation. However, Case 4, who did not receive carnitine supplementation, developed recurrent febrile seizures, suggesting that metabolic stress may exacerbate neurological vulnerability in untreated patients. This aligns with previous studies indicating that the risk of symptom manifestation increases during metabolic stressors, such as illness or prolonged fasting (Han et al., 2015). In this context, prophylactic L-carnitine supplementation may mitigate the risk of secondary carnitine depletion, metabolic crises, and associated neurological complications (Sass et al., 2004; Zhang et al., 2024). Given these potential risks, patient and caregiver education is essential. Families should be informed about metabolic stressors (e.g., fasting, infections) and the importance of early intervention during intercurrent illnesses, as prompt metabolic management may prevent acute decompensation.

Notably, some carriers of *ACAD8* gene variants have been observed to exhibit elevated C4-acylcarnitine levels (Oglesbee et al., 2007; Feng et al., 2021; Zhuang et al., 2021). This phenomenon has also been reported in population-based screenings, where multiple *ACAD8* heterozygotes were identified with metabolic abnormalities (Supplemental Table S5). The elevation of C4-acylcarnitine in carriers could stem from undetected genetic variants, such as deep intronic mutations or large-scale deletions/duplications, which may evade detection by NGS technology. Alternatively, a partial reduction in enzyme activity may impair isobutyryl-CoA metabolism, leading to C4 accumulation. In our study, one carrier presented with transient anemia, raising the question of whether certain heterozygotes might benefit from carnitine supplementation. Although carriers are generally asymptomatic, the presence of metabolic perturbations suggests that closer biochemical monitoring may be warranted, particularly during periods of metabolic stress such as illness or prolonged fasting.

This study broadens the known *ACAD8* mutation spectrum, identifies novel variants, and underscores the phenotypic variability of IBDD. Our findings highlight the need for early diagnosis via newborn screening, proactive carnitine supplementation, and long-term metabolic monitoring. Given the unpredictability of disease progression, structured follow-up and patient education are crucial to ensuring optimal clinical outcomes. However, this study is limited by its small sample size and relatively short follow-up duration. Larger, longitudinal studies are necessary to delineate the natural history of IBDD, refine genotype-phenotype correlations, and establish evidence-based management guidelines. Furthermore, functional characterization of the identified novel *ACAD8* variants is crucial to elucidating their pathogenic mechanisms and their impact on enzyme activity.

## Data Availability

The datasets presented in this study can be found in online repositories. The names of the repository/repositories and accession number(s) can be found in the article/Supplementary Material.
